# Structural determinants of child undernutrition: contribution of women’s empowerment in India’s nutritional transition

**DOI:** 10.1017/S1368980026103164

**Published:** 2026-07-20

**Authors:** Bharti Singh, Shri K. Singh, Deepanjali Vishwakarma

**Affiliations:** 1 Survey Research and Data Analytics, International Institute for Population Sciences, India; 2 School of Medicine, University of Limerick, Ireland

**Keywords:** Child nutrition, Underweight, Women’s empowerment, Socio-economic, NFHS, India

## Abstract

**Objective:**

This study aims to investigate the impact of women’s empowerment on child undernutrition from 2006 to 2021 in India at the state, primary sampling unit and household levels.

**Design:**

This study uses repeated cross-sectional data from three rounds of the National Family Health Survey (NFHS). Child undernutrition is assessed using stunting, wasting and underweight indicators, with women’s empowerment as a key predictor. Kernel density and linear polynomial plots depict the progression of anthropometric indicators over time, while multilevel regression identifies correlates and clustering in the undernutrition outcome.

**Participants:**

Children under 5 years of age from all twenty-eight states and nine union territories of India living with their biological mothers, who are currently married or in union and not pregnant, were included in the study.

**Results:**

Findings indicate that NFHS-5 reports the lowest prevalence of stunting and underweight, while severe wasting persists across all survey rounds. Women’s empowerment was not significant in NFHS-3, emerged as a significant determinant by NFHS-5 (NFHS-3 *β* = –0·08 (–0·26, 0·11); NFHS-5 *β* = –0·29** (–0·51, –0·07)). Among covariates, wealth quintile showed the strongest influence on child undernutrition from 2006 to 2021, with children from the richest households having the lowest risk of being underweight. Clustering remained higher at the household level in all survey rounds, reflected through the intra-class correlation.

**Conclusions:**

Higher women’s empowerment is significantly associated with reduced child undernutrition, where higher clustering persists at the household level. These findings highlight the need for targeted policies promoting women’s empowerment through community platforms and convergence with self-help groups to strengthen household-level health and nutrition outcomes.

Child undernutrition is a challenge in India despite substantial advances in economic development and public health interventions^(1)^. Globally, undernutrition in early childhood, characterised primarily by stunting, wasting and overweight, is a critical concern due to its lasting impact on physical and cognitive development, morbidity and mortality risks^(2–4)^. In India, which is home to nearly one-third of the world’s stunted children, addressing these challenges is critical for achieving the Sustainable Development Goals (SDG), particularly SDG 2 (Zero Hunger) and SDG 3 (Good Health and Well-being)^(5)^.

Women’s empowerment is a key social determinant of child health and nutritional outcomes. Empowered women are more likely to make autonomous decisions regarding healthcare, nutrition and childbearing practices, directly influencing their children’s overall health^(6)^. Empowerment, encompassing dimensions such as economic independence, decision-making autonomy, educational attainment and reduced exposure to intimate partner violence, has been shown to improve access to healthcare resources and enhance dietary diversity, which are critical factors for mitigating undernutrition among children^(7–9)^.

The relationship between women’s empowerment and child nutritional outcomes has been established through both theoretical and empirical perspectives, particularly in low- and middle-income countries, such as India^(9–11)^. Various literature indicate that empowering women across various dimensions not only enhances their health and agency but also significantly improves their children’s well-being^(12,13)^. The capability approach by Sen (1999)^(14)^, which conceptualises development as the expansion of individuals’ freedom to lead lives they value, provides a foundational understanding of how women’s agency contributes to child health. Empirical studies have emphasised the significance of structural inequalities, particularly in low- and middle-income countries^(15,16)^. A multi-country analysis by Smith *et al.* (2000) demonstrated that improvements in women’s status accounted for over half of the reductions in child malnutrition globally between 1970 and 1995. Several studies also have consistently shown that higher levels of maternal autonomy, particularly in decision-making and mobility, are positively associated with child height-for-age and dietary diversity^(9,11,17)^. In 2023, UNICEF reported that rural norms, early marriage, women’s employment and migration influence women’s nutritional knowledge and control over food allocation, thereby shaping child-feeding practices. Connell (1987) offers a critical perspective on the asymmetric power dynamics between genders that often hinder women from exerting control over household income, healthcare decisions and child-rearing practices^(18)^. In India, where deeply ingrained gender norms and intergenerational hierarchies persist, this theory of gender and power explains why financial inclusion alone may not lead to greater autonomy unless it is accompanied by gender equality^(19–21)^. For instance, ownership of land or bank account or working outside the home, often seen as indicators of empowerment, may not necessarily result in better child health outcomes if women lack the authority to utilise these resources^(22–25)^. This has been observed in several studies, which found that economic indicators of empowerment were less predictive of child nutrition than decision-making autonomy and freedom of mobility^(26)^. Furthermore, Crenshaw’s (1989)^(27)^ intersectionality theory provides a crucial understanding of the uneven distribution of empowerment and its outcomes across subgroups, aligning with the situation of Indian women. Women’s empowerment is not experienced uniformly among Indian women; rather, it is shaped by intersecting socio-economic identities, such as caste, religion, education level and geographic location. Several recent studies have confirmed the persistence of this intersectionality^(28,29)^. Furthermore, studies have found that the effect of maternal empowerment on child nutrition is significantly weaker among women from marginalised classes, even when controlling for education and income^(16,30,31)^. These findings are echoed in global systematic reviews. Pratley (2016)^(6)^ synthesised over sixty studies and concluded that maternal empowerment is a robust predictor of child nutritional outcomes, especially when measured through decision-making authority and educational attainment. Numerous studies have clearly shown that women’s empowerment is a critical, multidimensional driver of improved child nutrition outcomes. However, the effectiveness of this empowerment is contingent on context-specific factors^(9,13)^.

Despite numerous policy and programmatic interventions by governmental and non-governmental organisations, the prevalence of child undernutrition in India remains significantly high. Previous research often lags in providing a temporal analysis of child undernutrition in relation to women’s empowerment. Moreover, studies have typically measured women’s empowerment through a limited set of domains^(13,32,33)^. This study addresses this gap by analysing the National Family Health Survey (NFHS): NFHS-3, NFHS-4 and NFHS-5 data to assess trends in child undernutrition and to explore how a comprehensive six-domain women’s empowerment index correlates with undernutrition in children under 5 years of age. Furthermore, this study attempts to identify the key factors and clustering patterns that influence child undernutrition across India.

## Methods

### Data

The present study is based on the three most recent rounds of the National Family Health Survey (NFHS), which are cross-sectional surveys providing estimates of maternal and child health indicators, nutrition and women’s status at different levels. NFHS-3 (2005–2006), NFHS-4 (2015–2016) and NFHS-5 (2019–2021), also known as the Indian Demographic and Health Survey (DHS), were conducted by the International Institute for Population Sciences, Mumbai^(34–36)^. The NFHS dataset is hierarchical in nature, where individuals are nested within households, households within primary sampling units (PSU) and PSU within districts and states^(37)^. The NFHS sample is designed to provide national, state/union territory (UT) and district-level estimates of various indicators that are critical to monitor the SDG on population, health, nutrition and gender equality. However, some indicators, such as sexual behaviour, HIV/AIDS knowledge, attitudes and practices, domestic violence, men’s health and variables related to women’s empowerment (decision-making, attitude towards violence and mobility), are provided only at the state/UT level. The contents and definitions are similar to those of earlier rounds to allow for comparisons over time.

A stratified two-stage sampling design has been adopted in NFHS for collecting data on rural and urban areas. Within each rural stratum, villages were selected from the sampling frame using probability proportional to size, with explicit stratification based on the percentage of the SC/ST population and female literacy. The selection of households is based on the sampling frame prepared from the mapping and listing of households in all PSU. The NFHS uses four survey schedules – Household, Woman’s, Man’s and Biomarker – canvassed in local languages using the Computer-Assisted Personal Interviewing (CAPI) tool to provide estimates of population, health and family welfare indicators with a reasonable level of precision.

The NFHS-3 collected information from a nationally representative sample of 109 041 households, 124 385 women aged 15–49 years and 74 369 men aged 15–54 years. The NFHS-4 covered a nationally representative sample of 601 509 households, 699 686 women aged 15–49 years and 103 525 men aged 15–54 years in India^(38)^. The NFHS-5 covers 636 699 households with 724 115 eligible women aged 15–49 years from 30 456 PSU, comprising villages in rural areas and census enumeration blocks in urban areas. Hence, the study design is a repeated cross-sectional study, utilising three recent rounds of the NFHS. A total of 39 355 samples were selected in NFHS-3, 38 654 in NFHS-4 and 31 725 in NFHS-5 (see Table 1) for this study.

**Table 1.** Sample size of the children among under 5 years of age who were selected from this study Table 1 long description.

Selection criteria	NFHS-3 (2005–06)	NFHS-4 (2015–16)	NFHS-5 (2019–21)
Children aged 0–59 months included in the dataset	51 555	259 627	232 920
Children whose mothers were currently married and not pregnant	50 673	255 726	229 420
Children living with their biological mothers	48 223	253 973	227 694
Children whose mothers were interviewed for the women’s empowerment module	48 223	44 263	34 842
Children with valid anthropometric information (after excluding the missing values)	39 355	38 654	31 725

NFHS, National Family Health Survey.

### Variables

#### Dependent variable

The three anthropometric indicators used were height-for-age (HAZ or Stunting), weight-for-height (WHZ or Wasting) and weight-for-age (WAZ or Underweight) of 2 sd or more below the corresponding median of the reference population.

#### Independent variable

The independent variable of this study was women’s empowerment (low, middle and high). The index was constructed utilising twenty-seven variables under six dimensions: decision-making, attitude towards violence, freedom of movement, perceived sexual rights, financial independence and societal norms, using a second-order confirmatory factor analysis to generate the women’s empowerment index^(39)^. Details of the variables and dimensions are provided in the online supplementary material, Supplemental File (S1). Women were further classified into three categories based on tertiles of the empowerment score: those in the lowest tertile were categorised as low empowerment, those in the highest tertile as high empowerment and the remaining women in the middle tertile as middle empowerment^(40–42)^.

#### Covariates

The covariates included age of the child in months (2–6, 7–11, 12–17, 18–23, 24–34, 35–45 and 46–59), sex of the child (male and female), birth order (one, two to three, four and above), birth weight (small, average and above average), child anaemia (anaemic and non-anaemic), age of the mother in years (15–24, 25–34 and 35 and above), delivery by C-section (yes and no), institutional delivery (yes and no), ANC visits (less than four visits and four or more visits), occupation of the respondent (not in the workforce, agriculture and others), mother’s BMI (underweight, normal and overweight/obese), contraceptive use (yes and no), mother’s anaemia (anaemic and non-anaemic), wealth quintile (poorest, poorer, middle, richer and richest), caste (Schedule Caste/Schedule Tribe, Other Backward Class and Others), religion (Hindu, Muslim and others), place of residence (rural and urban) and region (Central, East, North, Northeast, South and West).

### Statistical analysis

The kernel density plots were used to understand the distribution of stunting, wasting and overweight, along with linear polynomial graphs, which facilitated an understanding of how these parameters evolve with the child’s age. Furthermore, multilevel modelling was applied considering the hierarchical structure of the data, where all the variables used for the different levels were discrete variables. A three-level random intercept logistic model was specified for the probability of an $i^{th}$ household in $j^{th}$ PSU and in the $k^{th}$ state reporting child undernutrition (Y_ijk_ = 1):

$$Logit\left(\pi_{ijk}\right)=\beta_{0}+\beta X_{ijk}+\left(\;f_{0k}+v_{0jk}+u_{0ij}\right)$$

This model estimates the log odds of $\pi_{ijk}$ adjusted for vector ( $X_{ijk}$ ) of the above-mentioned independent variables measured at the household level. The parameter $\beta_{0}$ represents the log odds of child undernutrition belonging to the reference category of all the categorical variables. The random effect inside the brackets is interpreted as a residual differential for state $k\;\left(f_{0k}\right)$ , PSU $j\;\left(v_{0jk}\right)$ and household $i\;\left(u_{0ij}\right)$ , which are assumed to be independent and normally distributed with mean 0 and variance $\sigma_{f_{0}}^{2}$ , $\sigma_{v_{0}}^{2}$ , and $\sigma_{u_{0}}^{2}$ , respectively^(43,44)^. These variances quantify the differences between states and PSU variations in the log odds of child undernutrition by adopting five models. Starting with the null model, which had no covariates, model 1 included child-related factors such as age of the child in months, sex of the child, birth order, birth weight, child anaemia status and women’s empowerment. Model 2 included mother-related variables such as women’s empowerment, age of the mother, delivery by C-section, institutional delivery, ANC visits, occupation of the respondent, mother’s BMI, contraceptive use and mother’s anaemia. Model 3 included household-related factors which are wealth quintile, caste, religion, place of residence and region, along with women’s empowerment. Model 4 contained all the covariates.

This study was analysed using STATA version 17 and R version 4.4.2. All results were derived by applying the sampling weight provided by the DHS, India.

## Results

Table 2 shows the distribution and variability in HAZ, WHZ and WAZ scores across NFHS 3, 4 and 5, with sd, variance, skewness and kurtosis providing insights into the data. The sd for all indicators has slightly declined, indicating reduced dispersion of child malnutrition levels. Likewise, decreased variance suggests greater uniformity in nutritional outcomes. Skewness values showed that WHZ was consistently right-skewed, with a higher concentration of normal weight-for-height children and a long tail of severely wasted cases. HAZ and WAZ had lower skewness, indicating a more symmetric distribution. The kurtosis for WHZ is above 3, indicating a leptokurtic distribution with more extreme values, while HAZ and WAZ have lower kurtosis, indicating a flatter dataset.

**Table 2.** Descriptive statistics of HAZ, WHZ and WAZ by NFHS-3, NFHS-4 and NFHS-5, India Table 2 long description.

Markers	NFHS-3			NFHS-4			NFHS-5		
	HAZ	WHZ	WAZ	HAZ	WHZ	WAZ	HAZ	WHZ	WAZ
Observation	46 655	46 655	46 655	219 796	219 796	219 796	201 276	197 314	205 641
Mean	0·480	0·198	0·425	0·383	0·210	0·357	0·354	0·193	0·320
sd	0·500	0·399	0·494	0·486	0·407	0·479	0·478	0·393	0·466
Variance	0·250	0·159	0·244	0·236	0·166	0·229	0·229	0·155	0·217
Skewness	0·079	1·510	0·303	0·480	1·420	0·598	0·609	1·560	0·770
Kurtosis	1·006	3·290	1·092	1·230	3·020	1·360	1·371	3·441	1·593

NFHS, National Family Health Survey.

**Figure 1.**
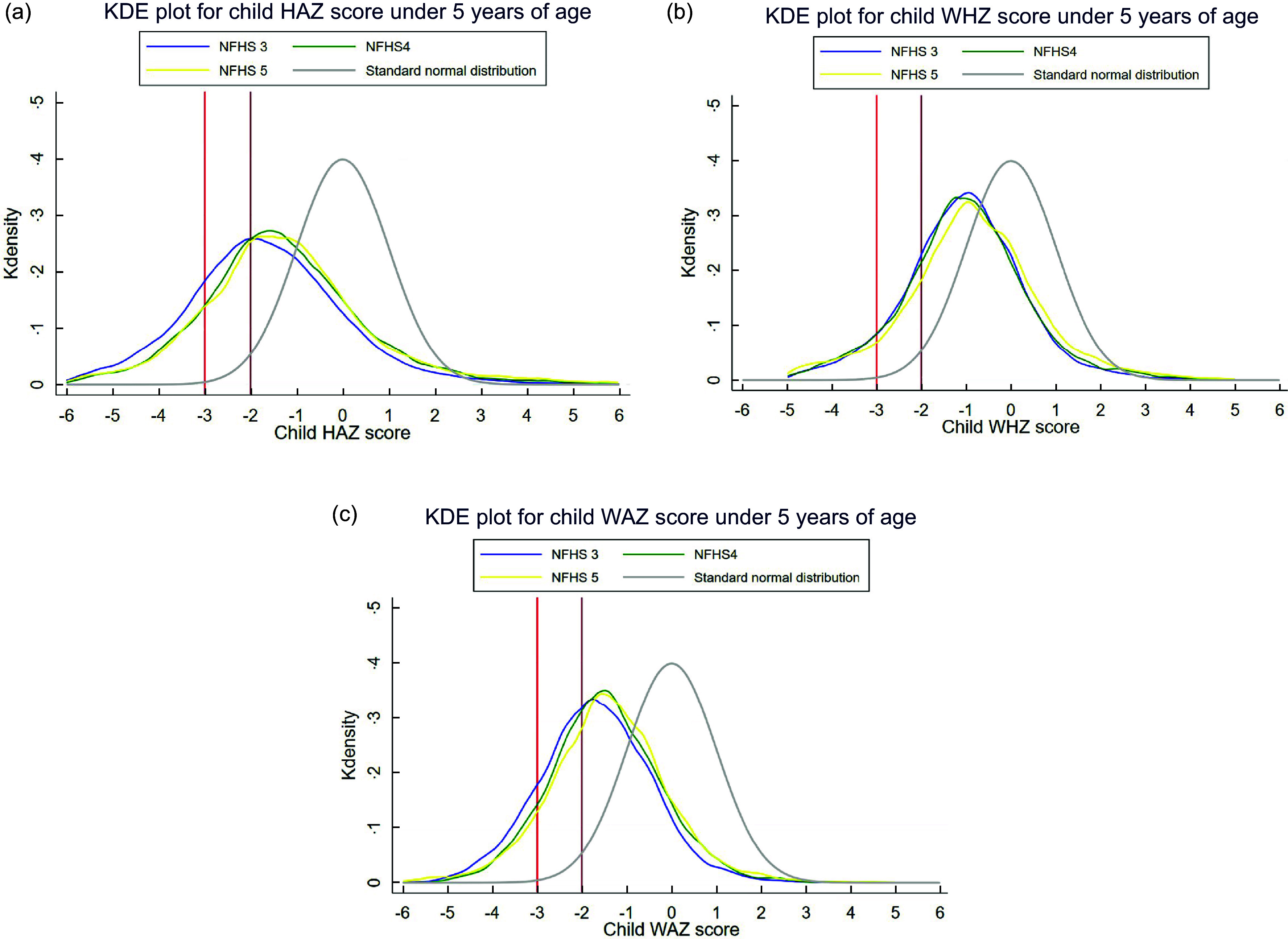
Figure 1 long description. (a) Kernel density plot for HAZ for children under 5 years of age, NFHS-3, NFHS-4 and NFHS-5, India. (b) Kernel density plot for WHZ for children under 5 years of age, NFHS-3, NFHS-4 and NFHS-5, India. (c) Kernel density plot for WAZ for children under 5 years of age, NFHS-3, NFHS-4 and NFHS-5, India. NFHS, National Family Health Survey.

Figure 1(a), (b) and (c) present the kernel density plot, revealing that the prevalence of stunting decreased from NFHS-3 to NFHS-5. While NFHS-4 and NFHS-5 had similar densities of severely stunted cases (below –3 sd from the median of the reference population), NFHS-5 showed a lower density of chronically undernourished cases (below –2 sd) than NFHS-4. Additionally, NFHS-5 indicated a limited decline in wasting among children under the age of 5 years compared to NFHS-3. The prevalence of severely wasted children remained consistent across NFHS 3, 4 and 5. Additionally, WHZ scores demonstrated a decline in acute and severely underweight cases (below –3 sd) from 2005–2006 to 2019–2021.

**Figure 2.**
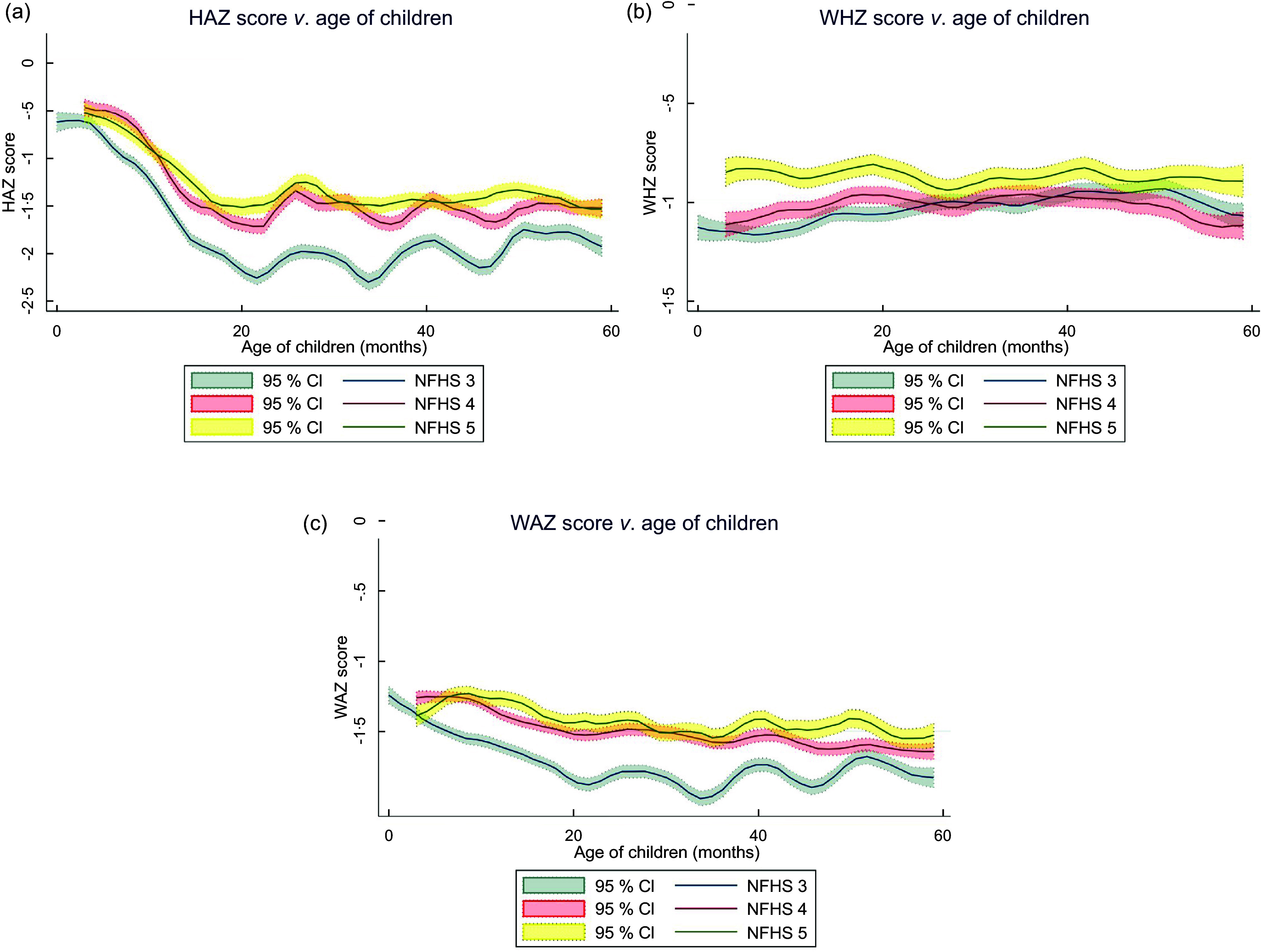
(a) Comparison of HAZ for children under 5 years of age, NFHS-3, NFHS-4 and NFHS-5, India. (b) Comparison of WHZ for children under 5 years of age, NFHS-3, NFHS-4 and NFHS-5, India. (c) Comparison of WAZ for children under 5 years of age, NFHS-3, NFHS-4 and NFHS-5, India. NFHS, National Family Health Survey.

Figure 2(a), (b) and (c) show a substantial decline in HAZ scores in early childhood, dropping from –0·5 sd to –2·3 sd among children aged 2 months to 20 months in NFHS-3. A similar trend was replicated in NFHS-4 and NFHS-5, although a smaller decline was observed in NFHS-5 compared to NFHS-3. Meanwhile, WHZ plots illustrated a steady pattern for children aged 0 to 5 years, with NFHS-3 showing lower WHZ scores and NFHS-5 exhibiting higher scores. WAZ scores experienced significant shifts during early childhood (0–20 months), declining to nearly –2 sd in NFHS-3 for children under the age of 20 months, while the reduction in WAZ scores with increasing age was less in NFHS-4 and NFHS-5.

Table 3 presents the results from multilevel regression models examining the association between women’s empowerment and undernutrition among children under 5 years of age in NFHS-3 (2005–2006), India. In the fixed-effect model, model 1, which adjusts for child-level characteristics, showed children of women with higher empowerment had a significantly 73 per cent lower likelihood of being undernourished. However, after controlling for mother-related variables, the magnitude of this effect reduced to 29 per cent for children of highly empowered women. In model 3, after adjusting for household-related variables, this effect started to diminish, with a reduced magnitude of –0·15*** (–0·24, –0·06).

**Table 3.** Multilevel regression results of undernourished children of under 5 years of age and women’s empowerment, NFHS-3 (2005–2006), India Table 3 long description.

	NFHS-3									
	Null model		Model 1		Model 2		Model 3		Model 4	
Covariates	*β*	95 % CI	*β*	95 % CI	*β*	95 % CI	*β*	95 % CI	*β*	95 % CI
Empowerment index										
Low ®										
Middle			−0·31***	–0·48, –0·13	−0·11*	–0·21, –0·01	−0·05	–0·14, 0·03	−0·03	–0·21, 0·15
High			−0·73***	–0·90, –0·57	−0·29***	–0·39, –0·19	−0·15***	–0·24, –0·06	−0·08	–0·26, 0·11
Child’s age										
2–6 months ®										
7–11 months			0·16	–0·39, 0·71					0·45	–0·07, 0·97
12–17 months			0·56*	0·02, 1·10					0·66*	0·14, 1·18
18–23 months			0·73*	0·19, 1·28					0·92**	0·38, 1·45
24–34 months			0·94***	0·40, 1·47					1·1***	0·57, 1·63
35–45 months			1·02***	0·49, 1·56					1·17***	0·63, 1·71
46–59 months			1·22***	0·69, 1·75					1·23***	0·69, 1·77
Child’s sex										
Female ®										
Male			−0·06	–0·16, 0·04					0·03	–0·08, 0·14
Birth order										
One ®										
Two to three			0·25***	0·14, 0·36					0·15*	0·01, 0·29
Four and above			0·61***	0·42, 0·80					0·33**	0·1, 0·55
Birth weight										
Small ®										
Normal or above			−1·04***	–1·19, –0·91					−0·77***	–0·96, –0·58
Child’s anaemia										
Non-anaemic®										
Anaemic			0·44***	0·32, 0·55					0·22***	0·09, 0·35
Mother’s age										
15–24years ®										
25–34 years					0·23***	0·15, 0·32			−0·04	–0·18, 0·1
35 years and above					0·49***	0·36, 0·63			0·02	–0·23, 0·27
Delivery by C-section										
No®										
Yes					−0·28***	–0·41, –0·15			−0·14	–0·30, 0·04
Institutional delivery										
No®										
Yes					−0·33***	–0·42, –0·23			−0·15	–0·33, 0·03
ANC visit										
Less than 4®										
Four or more					−0·28***	–0·37, –0·19			−0·08	–0·22, 0·06
Modern contraceptive use										
No®										
Yes					−0·02	–0·09, 0·06			−0·07	–0·2, 0·06
Mother’s BMI										
Underweight®										
Normal					−0·57***	–0·67, –0·48			−0·45***	–0·6, –0·3
Overweight					−1·08***	–1·23, –0·93			−0·82***	–1·03, –0·61
Mother’s anaemia										
Non-anaemic®										
Anaemia					0·1*	0·02, 0·17			−0·03	–0·15, 0·08
Mother’s occupation										
Not in workforce®										
Agriculture					0·29***	0·19, 0·39			0·01	–0·17, 0·2
Others					0·17**	0·06, 0·27			0·1*	–0·06, 0·26
Wealth quintile										
Poorest ®										
Poorer							−0·3***	–0·41, –0·19	−0·24	–0·53, 0·05
Middle							−0·67***	–0·78, –0·56	−0·42**	–0·7, –0·13
Richer							−1·14***	–1·26, –1·01	−0·79***	–1·11, –0·47
Richest							−1·92***	–2·07, –1·77	−1·24***	–1·62, –0·86
Caste										
SC/ST ®										
OBC							−0·2***	–0·29, –0·11	−0·24**	–0·41, –0·08
Others							−0·37***	–0·47, –0·28	−0·4***	–0·57, –0·22
Religion										
Hindu ®										
Muslim							0·16**	0·05, 0·27	0·22*	0·02, 0·42
Others							−0·3***	–0·43, –0·17	−0·15	–0·38, 0·08
Place of residence										
Urban ®										
Rural							−0·03	–0·12, 0·06	−0·16	–0·31, –0·02
Region										
North®										
Central							0·39	–0·14, 0·93	0·27	–0·27, 0·8
East							0·07	–0·35, 0·5	0·08	–0·36, 0·52
Northeast							−0·54**	–0·9, –0·18	−0·36	–0·76, 0·04
West							0·22	–0·24, 0·69	0·25	–0·2, 0·71
South							−0·33	–0·75, 0·09	−0·06	–0·48, 0·36
Variance										
State	0·38	0·22, 0·65	21·8	0·12, 0·41	0·15	0·08, 0·28	0·11	0·06, 0·19	0·08	0·04, 020
PSU	0·51	0·44, 0·60	37·72	0·26, 0·55	0·14	0·09, 0·22	0·21	0·17, 0·28	0·12	0·06, 0·34
Household	1·46	1·26, 1·70	1·86	1·37, 2·48	0·75	0·36, 1·58	1·32	1·13, 1·55	0·42	0·04, 5·02
ICC										
State	0·07	0·05, 0·13	0·04	0·02, 0·07	0·03	0·02, 0·06	0·02	0·01, 0·034	0·02	0·01, 0·04
PSU	0·16	0·13, 0·19	0·10	0·08, 0·14	0·07	0·05, 0·09	0·06	0·05, 0·08	0·05	0·03, 0·10
Household	0·41	0·38, 0·45	0·42	0·36, 0·49	0·24	0·15, 0·36	0·33	30, 0·37	0·16	0·03, 0·53

NFHS, National Family Health Survey; PSU, primary sampling unit.

*P < 0·05, **P < 0·01, ***P < 0·001.

After adjusting for all covariates in model 4, the effect of women’s empowerment on child undernutrition was no longer statistically significant. Among the adjusted covariates, children’s age and household economic status emerged as strong predictors of undernutrition. The likelihood of undernutrition increased progressively with age, with children aged 46–59 months showing significantly higher levels (1·23*** (0·69, 1·77)) compared to those aged 2–6 months. In contrast, higher wealth was strongly associated with lower child undernutrition (–1·24*** (–1·62, –0·86)).

In the random effect (Table 4), the null model (with no covariates) indicated that children aged under 5 years had a significant variation in the risk of undernutrition across PSU and household levels. The ICC coefficient suggested that a considerable proportion of the variation was attributed to differences between household (41 %) and PSU (16 %) in model 1. With the inclusion of covariates across successive models, the variance and ICC values declined substantially, particularly in model 4 (PSU ICC reduced from 0·16 to 0·06), indicating that the included covariates explained a significant proportion of the between-group variation. Table 4 presents the multilevel regression results examining the association between women’s empowerment and undernutrition among children under 5 years of age in NFHS-4 (2015–2016), India. The fixed-effect model indicated that women’s empowerment continued to be a significant predictor of child undernutrition, even after accounting for all covariates. Model 1 revealed that children of women with higher empowerment had a 1·13 times lower likelihood of undernourishment, followed by model 2, where the mother-related variables were adjusted (*β* = –1·10***; CI at 95 % (–1·28, –0·93)), and model 4, adjusting for all the covariates (*β* = –0·74***; CI 95 % (–0·98, –0·5)). After accounting for all the covariates in model 4, wealth status emerged as the most impactful factor in reducing undernutrition among children in NFHS-4. As wealth status increased, the risk of undernutrition decreased significantly. Additionally, the child’s birth weight (normal and above = –1·49*** (–1·73, –1·26)) and the mother’s BMI (obese and overweight = –1·74*** (–2, –1·48)) were protective factors for undernutrition in children. Moving towards the random effect (Table 5), the null model indicated substantial clustering in child undernutrition across different hierarchical levels. In this model, the maximum variation existed at the household level (ICC = 0·62 (0·60, 0·64)), followed by the PSU level (ICC = 0·12 (0·09, 0·16)). However, as the covariates were adjusted, the variation began to increase. In model 4, where all covariates were adjusted, the variation reached a maximum compared to all previous models, with maximum clustering at the household level (ICC = 0·79 (0·77, 0·81)). At the PSU and state levels, the ICC was nearly negligible, indicating minimal variation in child undernutrition.

**Table 4.** Multilevel regression results of undernourished children of under 5 years of age and women’s empowerment, NFHS-4 (2015–2016), India Table 4 long description.

Covariates	NFHS-4									
	Null model		Model 1		Model 2		Model 3		Model 4	
	*β*	95 % CI	*β*	95 % CI	*β*	95 % CI	*β*	95 % CI	*β*	95 % CI
Empowerment index										
Low ®										
Middle			−0·44***	–0·57, –0·3	−0·51***	–0·67, –0·36	−0·28***	–0·39, –0·17	−0·46***	–0·68, –0·24
High			−1·13***	–1·28, –0·99	−1·10***	–1·28, –0·93	−0·57***	–0·69, –0·44	−0·74***	–0·98, –0·5
Child’s age										
2–6 months ®										
7–11 months			−0·53*	–0·98, –0·09					−0·69	–1·32, –0·05
12–17 months			0·05	–0·4, 0·49					−0·06	–0·69, 0·56
18–23 months			0·39	–0·05, 0·83					0·52	–0·11, 1·15
24–34 months			0·46*	0·03, 0·89					0·85**	0·22, 1·47
35–45 months			0·62**	0·19, 1·05					0·84**	0·2, 1·47
46–59 months			0·56*	0·13, 0·99					0·78*	0·15, 1·42
Child’s sex										
Female ®										
Male			0·1*	0, 0·2					0·24**	0·07, 0·4
Birth order										
One ®										
Two to three			0·29***	0·18, 0·39					0·29**	0·08, 0·49
Four and above			0·68***	0·5, 0·86					0·53**	0·19, 0·86
Birth size										
Small ®										
Normal and above			−1·06***	–1·19, –0·92					−1·49***	–1·73, –1·26
Child’s anaemia										
Non-anaemic®										
Anaemic			0·46***	0·36, 0·57					0·58***	0·4, 0·76
Mother’s age										
15–24 years ®										
25–34 years					0·24***	0·1, 0·38			−0·08	–0·29, 0·13
35 years and above					0·65***	0·42, 0·88			0·1	–0·26, 0·47
Delivery by C-section										
No®										
Yes					−0·54***	–0·72, –0·36			−0·36***	–0·59, –0·14
Institutional delivery										
No®										
Yes					−0·22***	–0·39, –0·05			0·02	–0·32, 0·36
ANC visit										
Less than 4®										
Four or more					−0·14	–0·29, 0			0·05	–0·14, 0·24
Contraceptive use										
No®										
Yes					0·12	–0·01, 0·26			−0·08	–0·26, 0·11
Mother’s BMI										
Underweight®										
Normal					−0·93***	–1·09, –0·78			−1·00***	–1·21, –0·78
Overweight/obese					−1·86***	–2·05, –1·66			−1·74***	–2, –1·48
Mother’ anaemia										
Non-anaemic®										
Anaemic					0·18**	0·05, 0·31			0·14	–0·03, 0·31
Mother’s occupation										
Not in workforce®										
Agriculture					0·32***	0·12, 0·51			−0·08	–0·35, 0·19
Other					0·34***	0·13, 0·55			0·33*	0·06, 0·61
Wealth quintile										
Poorest ®										
Poorer							−0·48***	–0·61, –0·35	−0·62***	–0·91, –0·33
Middle							−0·97***	–1·12, –0·83	−1·2***	–1·52, –0·89
Richer							−1·46***	–1·64, –1·29	−1·82***	–2·17, –1·46
Richest							−2·02***	–2·23, –1·82	−2·22***	–2·63, –1·82
Caste										
SC/ST ®										
OBC							−0·23***	–0·34, –0·12	−0·19	–0·4, 0·02
Others							−0·56***	–0·71, –0·42	−0·54***	–0·8, –0·28
Religion										
Hindu ®										
Muslim							0·14*	0, 0·29	0·07	–0·21, 0·36
Others							−0·02	–0·27, 0·22	−0·01	–0·42, 0·4
Place of residence										
Urban ®										
Rural							−0·16*	–0·29, –0·03	−0·27*	–0·49, –0·05
Region										
North®										
Central							0·45	–0·03, 0·93	0·66*	0·1, 1·21
East							0·05	–0·39, 0·48	0·04	–0·47, 0·54
Northeast							−1·11***	–1·59, –0·62	−0·96	–1·61, –0·31
West							0·48	–0·01, 0·96	0·48**	–0·07, 1·02
South							−0·23	–0·64, 0·18	0·07	–0·41, 0·55
Variance										
State	0·56	0·32, 0·97	0·38	0·21, 0·68	0·30	0·15, 0·56	0·10	0·05, 0·21	0·10	0·04, 0·24
PSU	0·50	0·39, 0·65	0·49	0·32, 0·75	0·26	0·10, 0·72	0·28	0·18, 0·44	0·35	0·29, 0·46
Household	4·24	3·92, 4·59	5·51	4·99, 6·07	8·26	7·39, 9·23	4·30	3·96, 4·67	12·28	10·63, 14·21
ICC										
State	0·06	0·34, 0·10	0·04	0·01, 0·02	0·02	0·01, 0·04	0·01	0·01, 0·03	0·01	0·002, 0·016
PSU	0·12	0·09, 0·16	0·09	0·06, 0·12	0·05	0·02, 0·08	0·05	0·03, 0·07	0·01	0·003, 0·016
Household	0·62	0·60, 0·64	0·66	0·64, 0·68	0·73	0·71, 0·75	0·59	0·57, 0·61	0·79	0·77, 0·81

NFHS, National Family Health Survey; PSU, primary sampling unit.

*P < 0·05, **P < 0·01, ***P < 0·001.

**Table 5.** Multilevel regression results of undernourished children of under 5 years of age and women’s empowerment, NFHS-5 (2019–2021), India

	NFHS-5									
	Null model		Model 1		Model 2		Model 3		Model 4	
Covariates	*β*	95 % CI	*β*	95 % CI	*β*	95 % CI	*β*	95 % CI	*β*	95 % CI
Empowerment index										
Low ®										
Middle			−0·21**	–0·35, –0·07	−0·23*	–0·40, –0·05	−0·10	–0·23, 0·02	−0·19*	–0·40, 0·02
High			−0·5***	–0·64, –0·35	−0·58***	–0·76, –0·39	−0·26***	–0·4, –0·13	−0·29**	–0·51, –0·07
Child’s age										
2–6 months ®										
7–11 months			−0·66*	–1·17, –0·15					−0·75*	–1·46, –0·04
12–17 months			−0·11	–0·61, 0·4					−0·28	–0·98, 0·43
18–23 months			0·11	–0·39, 0·61					0·11	–0·6, 0·81
24–34 months			0·25	–0·24, 0·74					0·40	–0·30, 1·10
35–45 months			0·25	–0·25, 0·74					0·32*	–0·38, 1·03
46–59 months			0·34	–0·15, 0·83					0·35*	–0·36, 1·06
Child’s sex										
Female ®										
Male			0·28***	0·18, 0·38					0·44***	0·27, 0·61
Birth order										
One ®										
Two to three			0·39***	0·29, 0·5					0·63***	0·43, 0·84
Four and above			0·79***	0·61, 0·98					0·89***	0·55, 1·23
Birth weight										
Small ®										
Normal and above-average			−1·01***	–1·15, –0·88					−1·37***	–1·6, –1·14
Child’s anaemia										
Non-anaemic®										
Anaemic			0·44***	0·33, 0·55					0·55***	0·36, 0·74
Mother’s age										
15–24 years ®										
25–34 years					0·3***	0·14, 0·47			0·09	–0·13, 0·3
35 years and above					0·34*	0·07, 0·61			−0·31	–0·67, 0·05
Delivery by C-section										
No®										
Yes					−0·57***	–0·76, –0·39			−0·25*	–0·47, –0·03
Institutional delivery										
No®										
Yes					−0·36***	–0·6, –0·11			−0·14	–0·51, 0·24
ANC visit										
Less than 4®										
Four or more					−0·22**	–0·37, –0·06			−0·09	–0·27, 0·1
Contraceptive use										
No®										
Yes					0·14	–0·01, 0·3			0·06	–0·14, 0·25
Women’s BMI										
Underweight®										
Normal					−0·68***	–0·87, –0·49			−0·59***	–0·81, –0·37
Overweight/obese					−1·45***	–1·67, –1·23			−1·36***	–1·62, –1·1
Mother’s anaemia										
Non-anaemic®										
Anaemic					0·14	0, 0·29			0·04	–0·14, 0·21
Mother’s occupation										
Not in workforce®										
Agriculture					0·48***	0·25, 0·71			0·14	–0·12, 0·41
Others					0·16	–0·39, 0·07			−0·33*	–0·6, –0·07
Wealth quintile										
Poorest ®										
Poorer							−0·55***	–0·7, –0·4	−0·45***	–0·72, –0·18
Middle							−0·83***	–1, –0·66	−0·70***	–0·99, –0·41
Richer							−1·24***	–1·43, –1·05	−0·96***	–1·28, –0·63
Richest							−1·8***	–2·03, –1·57	−1·60***	–1·98, –1·22
Caste										
SC/ST ®										
OBC							−0·24***	–0·37, –0·12	−0·38***	–0·58, –0·17
Others							−0·59***	–0·76, –0·42	−0·74***	–1·01, –0·47
Religion										
Hindu ®										
Muslim							0·16	–0·02, 0·33	0·34*	0·05, 0·62
Others							−0·27	–0·55, 0·01	−0·42	–0·85, 0·02
Place of residence										
Urban ®										
Rural							−0·02	–0·17, 0·13	−0·12	–0·35, 0·12
Region										
North®										
Central							0·36	–0·08, 0·8	0·51*	0·01, 1·01
East							0·48*	0·06, 0·89	0·84***	0·38, 1·31
Northeast							−0·42	–0·9, 0·05	0·04	–0·57, 0·65
West							1·10***	0·61, 1·59	1·47***	0·92, 2·01
South							0·13	–0·27, 0·52	0·59	0·12, 1·05
Variance										
State	0·44	0·24, 0·77	0·41	0·22, 0·72	0·30	0·16, 0·57	0·083	0·04, 0·18	0·07	0·03, 0·20
PSU	0·74	0·56, 0·93	0·73	0·54, 0·99	0·69	0·39, 1·22	0·54	0·39, 0·74	0·42	0·11, 1·56
Household	4·66	4·25, 5·10	5·20	4·69, 5·77	9·95	8·63, 11·48	4·60	4·18, 5·05	11·03	9·21, 13·21
ICC										
State	0·05	0·03, 0·08	0·04	0·02, 0·07	0·02	0·01, 0·04	0·01	0·01, 0·02	0·01	0·002, 0·014
PSU	0·13	0·10, 0·16	0·12	0·08, 0·15	0·07	0·04, 0·11	0·07	0·06, 0·10	0·03	0·02, 0·10
Household	0·64	0·61, 0·66	0·66	0·64, 0·68	0·77	0·75, 0·79	0·61	0·59, 0·63	0·78	0·75, 0·80

NFHS, National Family Health Survey; PSU, primary sampling unit; ICC is intraclass correaltion.

*P < 0·05, **P < 0·01, ***P < 0·001.

Table 5 presents the multilevel regression results examining the association between women’s empowerment and undernutrition among children under 5 years of age in NFHS-5 (2019–2021), India. The fixed-effects models indicate that women’s empowerment remained a significant determinant of child undernutrition across most model specifications. The completely adjusted model (model 4) revealed that children of higher-empowered mothers were 0·30 times less likely to experience undernutrition.

Among the covariates, wealth status remained the dominant predictor of child undernutrition from 2006 to 2021. In NFHS-5, children from economically well-off households were 1·6 times less likely to be undernourished. Another consistent factor affecting undernutrition in children was birth weight. Children who were born with normal or above birth weight had a 1·4 times lower risk of undernourishment. The sex of the child also showed a statistically significant effect, with male children being more prone to undernutrition (*β* = 0·44 *** (0·27, 0·61)). Furthermore, children suffering from anaemia have a one-in-two chance of being undernourished.

The random effects model (see Table 5) showed a consistent pattern with previous survey results, indicating that household-level clustering is more pronounced than clustering at the PSU and state levels. Furthermore, as the covariates were included in the models, the variation in the PSU and state level began to diminish. In contrast, household-level clustering remained substantial and increased in the fully adjusted model (ICC = 0·78; (0·75, 0·80)), suggesting that unobserved household-level factors continued to account for a substantial proportion of the variation in child undernutrition. This highlights the persistent importance of household context in shaping nutritional outcomes, even after accounting for a wide range of individual, maternal and socio-economic characteristics.

## Discussion

This study explored the relationship between women’s empowerment and child undernutrition in India from 2006 to 2021, using NFHS data. By utilising multilevel modelling, our findings provide insights into the evolving patterns of stunting, wasting and overweight among children under 5 years of age and the significant role of maternal empowerment in mitigating undernutrition.

Our results portray that while stunting has gradually declined across NFHS-3, NFHS-4 and NFHS-5, severe stunting cases remained consistent between NFHS-4 and NFHS-5. This suggests that, although there have been overall improvements, the most vulnerable populations continue to face chronic undernutrition. The limited decline in wasting observed in the NFHS-5 further highlights the persistent challenge of acute malnutrition, which remains a major concern despite various policy interventions targeting child nutrition and suggests structural issues at the community and household levels^(36,45)^. A recent study confirmed that household characteristics significantly influence child nutrition outcomes, emphasising the importance of targeting household-level determinants to prevent poor child growth^(46)^.

A key finding of this study was the significant association between women’s empowerment and the risk of lower child undernutrition. Children of highly empowered mothers had lower likelihood of being undernourished even after adjusting for socio-economic and demographic variables. However, the strength of this association varied across the survey years. In the NFHS-3, the effect of women’s empowerment on child undernutrition diminished after controlling for household- and community-level factors, suggesting that broader socio-economic determinants played a dominant role in child nutrition during this period. In contrast, women’s empowerment remained significantly associated with child undernutrition in NFHS-4 and NFHS-5. This association persisted even after adjusting for all covariates, indicating its growing importance as a determinant of child health over time. This aligns with findings from a study across East African countries, which highlighted the positive influence of women’s intrinsic agency on maternal and child nutrition^(13)^. Additionally, a 2020 study conducted in East African countries found that women’s intrinsic agency positively influences their maternal and child nutritional status, suggesting that enhancing women’s decision-making autonomy can lead to better child nutrition^(47)^.

These findings affirm the centrality of Empowerment Theory in understanding how women’s autonomy, especially in decision-making, financial control and mobility, translates into improved child health outcomes. As theorised by Kabeer (1999) and Malhotra & Schuler (2005), empowered women are better positioned to allocate resources, seek timely healthcare and ensure nutritional adequacy for their children^(48,49)^.

Multilevel analysis further underscores the role of household-level factors in shaping child nutritional outcomes. Across all survey years, household-level clustering was the most prominent, with intra-class correlation coefficients, particularly in the NFHS-4 and NFHS-5. This suggests that household characteristics such as economic status, maternal education and household decision-making dynamics significantly influence child undernutrition. Wealth status emerged as the most consistent predictor, with children from wealthier households having a substantially lower likelihood of developing undernutrition. A recent analysis of NFHS-5 highlighted key predictors of undernutrition, such as maternal access to financial and communication resources, emphasising how strengthening women’s empowerment can positively impact children’s nutritional status^(50)^. These household-level dynamics reflect the core principles of Human Capital Theory, which posits that investments in maternal education and skills yield long-term returns on better child health outcomes^(14,51)^.

Further, the findings also highlight gender-based disparities, with male children being more susceptible to undernutrition than female children. This aligns with previous studies suggesting that male children may have higher energy requirements or greater vulnerability to infections, which could contribute to higher undernutrition rates. From a broader lens, these results also align with the Public Health Development framework, which emphasises the structural determinants of health and the intersectoral nature of solutions. The persistent role of socio-economic and household factors even in the presence of national nutrition programmes signals the need for public health approaches that incorporate gender empowerment, poverty alleviation and education as integrated strategies to combat undernutrition^(15,52)^.

Our study offers critical insights for policy and programmatic action aimed at reducing child undernutrition in India. Strengthening women’s empowerment, particularly by enhancing decision-making autonomy, financial independence and mobility, is a vital pathway for improving child nutrition outcomes. Initiatives that expand maternal education, create economic opportunities for women and improve their access to quality healthcare can significantly impact child growth and well-being. Simultaneously, the strong influence of household-level factors highlights the need for targeted household-focused interventions. Direct nutritional support, income assistance and gender-sensitive nutritional programmes tailored to household dynamics can help address persistent undernutrition. Furthermore, the observed gender disparities call for child nutrition strategies that acknowledge and respond to the specific vulnerabilities of male children, thereby ensuring equitable outcomes across genders.

### Limitations

Despite these valuable insights, this study has some limitations. First, while our analysis established associations, it did not imply causation. Future research should explore longitudinal data to examine the causal pathways between women’s empowerment and child nutrition. Second, while we controlled for several socio-economic and demographic factors, unobserved confounders, such as cultural practices and intra-household food distribution patterns, may influence child nutrition outcomes. Finally, our study primarily relied on NFHS survey data, which, despite its robustness, may be subject to recall bias and reporting inaccuracies.

### Conclusions

In conclusion, this study highlights the critical role of women’s empowerment in improving child nutritional outcomes in India. This study concludes that while the association between women’s empowerment and child undernutrition was reduced after adjusting for covariates in NFHS-3 (2005–2006), it remained statistically significant in NFHS-4 (2015–2016) and NFHS-5 (2019–2021), indicating an independent role of women’s empowerment over time. At the same time, household socio-economic conditions continue to be strong determinants of undernutrition, and a substantial proportion of variation in nutritional outcomes persists at the household level, suggesting that interventions to reduce child undernutrition in India should place greater emphasis at the household level. As India continues its efforts to meet the SDG, particularly SDG 2 (Zero Hunger) and SDG 3 (Good Health and Well-being), policy approaches that integrate gender-based empowerment with household-level and broader socio-economic interventions will be essential for achieving sustained progress in reducing undernutrition in children. Policies and programmes aimed at enhancing women’s empowerment at the community level should be designed leveraging community platforms and convergence with programmes and activities of self-help groups and Panchayati raj Institution, where women may have the opportunity to transition from proxy to policymakers and implementers of various health and nutrition programmes.

## Supporting information

10.1017/S1368980026103164.sm001 Singh et al. supplementary material Singh et al. supplementary material

## Supplementary material

For supplementary material accompanying this paper visit https://doi.org/10.1017/S1368980026103164

## Table 1. Long description

A table comparing the sample sizes of children under 5 years of age selected from three rounds of the National Family Health Survey in India. The table has 5 rows and 3 columns. The columns are labeled NFHS-3 (2005-06), NFHS-4 (2015-16), and NFHS-5 (2019-21). The rows are labeled with different selection criteria. Row 1: Children aged 0-59 months included in the dataset. NFHS-3: 51555, NFHS-4: 259627, NFHS-5: 232920. Row 2: Children whose mothers were currently married and not pregnant. NFHS-3: 50673, NFHS-4: 255726, NFHS-5: 229420. Row 3: Children living with their biological mothers. NFHS-3: 48223, NFHS-4: 253973, NFHS-5: 227694. Row 4: Children whose mothers were interviewed for the women’s empowerment module. NFHS-3: 48223, NFHS-4: 44263, NFHS-5: 34842. Row 5: Children with valid anthropometric information (after excluding the missing values). NFHS-3: 39355, NFHS-4: 38654, NFHS-5: 31725.

Navigate back to Table 1..

## Table 2. Long description

A table comparing descriptive statistics of HAZ, WHZ, and WAZ scores across NFHS-3, NFHS-4, and NFHS-5 in India. The table has 8 rows and 12 columns. The columns are labeled as Markers, Observation, Mean, sd, Variance, Skewness, and Kurtosis for each of the NFHS-3, NFHS-4, and NFHS-5. The row labels are HAZ, WHZ, and WAZ. Row 1: Markers, Observation, 46 655, 46 655, 46 655, 219 796, 219 796, 219 796, 201 276, 197 314, 205 641. Row 2: Mean, 0.480, 0.198, 0.425, 0.383, 0.210, 0.357, 0.354, 0.193, 0.320. Row 3: sd, 0.500, 0.399, 0.494, 0.486, 0.407, 0.479, 0.478, 0.393, 0.466. Row 4: Variance, 0.250, 0.159, 0.244, 0.236, 0.166, 0.229, 0.229, 0.155, 0.217. Row 5: Skewness, 0.079, 1.510, 0.303, 0.480, 1.420, 0.598, 0.609, 1.560, 0.770. Row 6: Kurtosis, 1.006, 3.290, 1.092, 1.230, 3.020, 1.360, 1.371, 3.441, 1.593.

Navigate back to Table 2..

## Figure 1. Long description

Three kernel density plots depict child undernutrition scores under 5 years of age in India across different surveys. Panel A: Kernel density plot for child HAZ score under 5 years of age. The x-axis represents the Child HAZ score ranging from -6 to 6, and the y-axis represents the density. The plot includes data from NFHS 3, NFHS 4, NFHS 5, and a standard normal distribution. The curves show the distribution of HAZ scores, with NFHS 3 in blue, NFHS 4 in green, NFHS 5 in yellow, and the standard normal distribution in gray. Panel B: Kernel density plot for child WHZ score under 5 years of age. The x-axis represents the Child WHZ score ranging from -6 to 6, and the y-axis represents the density. The plot includes data from NFHS 3, NFHS 4, NFHS 5, and a standard normal distribution. The curves show the distribution of WHZ scores, with NFHS 3 in blue, NFHS 4 in green, NFHS 5 in yellow, and the standard normal distribution in gray. Panel C: Kernel density plot for child WAZ score under 5 years of age. The x-axis represents the Child WAZ score ranging from -6 to 6, and the y-axis represents the density. The plot includes data from NFHS 3, NFHS 4, NFHS 5, and a standard normal distribution. The curves show the distribution of WAZ scores, with NFHS 3 in blue, NFHS 4 in green, NFHS 5 in yellow, and the standard normal distribution in gray.

Navigate back to Figure 1..

## Table 3. Long description

The table presents multilevel regression results examining the association between women’s empowerment and undernutrition among children under 5 years of age in India during 2005-2006. The table includes four models: Null model, Model 1, Model 2, and Model 3, each adjusting for different sets of variables. The columns are labeled with the models and include the coefficients and 95 percent confidence intervals for various covariates. Row labels include variables such as Empowerment index, Child’s age, Child’s sex, Birth order, Birth weight, Child’s anemia, Mother’s BMI, Mother’s age, Delivery by C-section, Institutional delivery, ANC visit, Modern contraceptive use, Mother’s occupation, Wealth quintile, Caste, Religion, Place of residence, and Region. Each row provides the coefficients and confidence intervals for the respective covariates across the different models. Notable trends include the reduction in the magnitude of the effect of women’s empowerment on child undernutrition as more variables are controlled for in subsequent models.

Navigate back to Table 3..

## Table 4. Long description

A table with 45 rows and 11 columns showing multilevel regression results of undernourished children under 5 years of age and women’s empowerment in India. The columns are Covariates, Null model β, Null model 95% CI, Model 1 β, Model 1 95% CI, Model 2 β, Model 2 95% CI, Model 3 β, Model 3 95% CI, Model 4 β, and Model 4 95% CI. The rows include various covariates such as Empowerment index, Child’s age, Child’s sex, Birth order, Birth size, Child’s anemia, Mother’s age, Delivery by C-section, Institutional delivery, ANC visit, Contraceptive use, Mother’s BMI, Mother’s anemia, Mother’s occupation, Wealth quintile, Caste, Religion, Place of residence, and Region. Each row provides the regression coefficients (β) and their 95% confidence intervals (CI) for the null model and four different models. Notable trends include the significant impact of wealth status on reducing undernutrition among children, with higher wealth status associated with a lower risk of undernutrition. Additionally, the child’s birth weight and the mother’s BMI are identified as protective factors against undernutrition.

Navigate back to Table 4..
