# Supplementary material for: Structural determinants of child undernutrition: contribution of women’s empowerment in India’s nutritional transition
Source: Public Health Nutr. 2026 Jul 20;29(1):e167. doi: 10.1017/S1368980026103164 (PMC13469224; doi:10.1017/S1368980026103164)
Supplement: Supplementary file 1 [file S1368980026103164sup001.docx]

Supplementary 1: Description of the domain and variables of women’s empowerment

| **Domain** | **Variables** | **Score** |  |
| --- | --- | --- | --- |
| **Attitude towards Violence** | | |  |
|  | Whether beating is justified if the wife goes out without telling the husband | Justified=0, not justified=1 |  |
|  | Whether beating is justified if the wife neglects the children | Justified=0, not justified=1 |  |
|  | Whether beating is justified if the wife argues with the husband | Justified=0, not justified=1 |  |
|  | Whether beating is justified if the wife refuses to have sex with the husband | Justified=0, not justified=1 |  |
|  | Whether beating is justified if the wife burns the food | Justified=0, not justified=1 |  |
| **Decision-Making** | | |  |
|  | Who Decides on the respondent's healthcare? | Decides alone or with partner=1, else =0 |  |
|  | Who Decides on large household purchases? | Decides alone or with partner=1, else =0 |  |
|  | Who Decides on visits to family or relatives? | Decides alone or with partner=1, else =0 |  |
|  | Who Deciding what to do with the money the husband earns? | Decides alone or with partner=1, else =0 |  |
| **Freedom of Mobility** | | |  |
|  | Are you allowed to go to the market? | Allowed to go alone or with someone, else=0 |  |
|  | Are you allowed to go to the health facility? | Allowed to go alone or with someone=1, else=0 |  |
|  | Are you allowed to go to the village? | Allowed to go alone or with someone=1, else=0 |  |
| **Perceived Sexual Rights** | | |  |
|  | Refused to have sex if the wife knows her husband has sex with other women | Yes=1, No=0 |  |
|  | Say no to having sex if the wife does not want to have sexual intercourse. | Yes=1, No=0 |  |
|  | Refused to have sex if the wife knows her husband has a sexually transmitted disease | Yes=1, No=0 |  |
| **Financial Security** | | |  |
|  | Do you have a bank account? | Yes=1, No=0 |  |
|  | Do you own a house? | Yes=1, No=0 |  |
|  | Do you have a mobile phone? | Yes=1, No=0 |  |
|  | Do you own a land? | Yes=1, No=0 |  |
|  | Have you worked in the last 12 months? | Yes=1, No=0 |  |
|  | Do you have any money of your own that you alone can decide how to use? | Yes=1, No=0 |  |
| **Societal Norm** | | |  |
|  | Use Media exposure (read, radio and TV) | Yes=1, No=0 |  |
|  | Women's education | Secondary and higher=1, No education or primary=0 |  |
|  | Age of respondent at cohabitation | 18 and above=1, else=0 |  |
|  | Age of respondent at first birth | 18 and above=1, else=0 |  |
|  | Age difference: woman’s minus husband’s age | Equal or greater=1, else 0 |  |
|  | Education difference: woman’s minus husband’s years of schooling | Equal or greater=1, else 0 |  |
